# Exploring the impact of subjective well-being on medication adherence: A cross-sectional study among individuals with multiple chronic diseases

**DOI:** 10.1016/j.rcsop.2024.100496

**Published:** 2024-08-25

**Authors:** Mohamad Ismail, Mayssah El-Nayal, Souraya Domiati

**Affiliations:** a Department of Pharmacy Practice, Faculty of Pharmacy, Beirut Arab University, Beirut, Lebanon; b Department of Psychology, Faculty of Human Sciences, Beirut Arab University, Beirut, Lebanon; c Department of Pharmacology and Therapeutics, Faculty of Pharmacy, Beirut Arab University, Beirut, Lebanon

**Keywords:** Chronic diseases, Medication adherence, ARMS-A, Subjective well-being, Lebanon

## Abstract

**Background:**

Medication non-adherence is a significant barrier to optimal treatment goals. The study explores the association between subjective well-being (SWB) and medication adherence among Lebanese individuals with multiple chronic diseases and identifies additional factors that may influence adherence in this population.

**Methods:**

An exploratory, cross-sectional study was conducted for three months at six community pharmacies. Adherence was assessed using the Adherence to Refills and Medication Scale Arabic Lebanese Version (ARMS-A). The SWB was measured using the Arabic Scale of Happiness (ASH), Love of Life Scale (LLS), Arab Hope Scale (AHS), and Satisfaction with Life Scale (SWLS). Spearmen's Rho correlation analyzed the association between ARMS-A and SWB constructs. Binary logistic regression identified predictors of adherence among individuals with chronic diseases and on multiple chronic medications.

**Results:**

Of 400 participants, 106 (26.5 %) with a 95 % CI, 0.22–0.31, were adherent. Lower medication adherence (reflected in higher ARMS-A scores) was associated with lower SWB (*p* = 0.01). Multivariate analysis showed that lower education (OR = 2.21, 95 % CI, 1.01–4.81), lack of a specific diet (OR = 1.64, 95 % CI, 1.01–2.69), and frequent hospital and/or emergency visits (OR = 3.29, 95 % CI, 1.75–6.17 for 2 visits; OR = 2.71, 95 % CI, 1.43–5.14 for ≥3 visits) significantly increased the odds of non-adherence to chronic treatment. However, higher income (OR = 0.06, 95 % CI, 0.01–0.38), healthcare provider occupation (OR = 0.42, 95 % CI, 0.21–0.48), and having diabetes mellitus (OR = 0.59, 95 % CI, 0.36–0.96) correlated with better adherence.

**Conclusion:**

A significant portion of participants failed to adhere to their prescribed chronic medications, influenced by multicomplex socioeconomic, psychological, and health-related factors. These findings demonstrate the need for culturally-tailored, pharmacist-led interventions to improve medication adherence and overall health outcomes.

## 1 Introduction

The shifts in population characteristics, such as aging, environmental factors, and genetic predisposition, occurring in the twenty-first century in both developed and developing countries are causing an increase in the frequency of chronic diseases and subsequently becoming the leading cause of death worldwide.^1^ This rise is further exacerbated by numerous factors including behavioral, psychosocial, and societal.^1^ Consequently, pharmacological therapies have become essential in managing these chronic diseases. Yet, about 50 % of individuals with chronic diseases do not take their chronic medications as prescribed.^2^

Medication adherence, defined as “the extent to which a person's behavior – taking medication, following a diet, and/or executing lifestyle changes – corresponds with agreed recommendations from a healthcare provider”,^3^ is vital for effective treatment. Non-adherence behavior is linked with a range of negative consequences including waste of medication and money, disease progression, poor clinical outcomes, increased hospital admissions, and even death.^4^ Recognizing the complexity of medication adherence, the World Health Organization has developed five dimensions that shape the individual's capability to properly adhere to the recommended therapy, including social and economic, healthcare provider and healthcare system, condition-related, therapy-related, and patient-related factors.^2^ Since then, ameliorating medication non-adherence has become a predominant topic on the public health agenda.

To address this topic, researchers are exploring new approaches to improve medication adherence. One promising psychological model is positive psychology, defined as “scientific study of what makes life most worth living”.^5^^,^^6^ According to Fredrickson, 2001, positive emotional experiences can stretch the person's cognitive and behavioral capabilities, and potentially promote health behaviors^7^ and treatment adherence.^8^^,^^9^ In contrast, negative emotions can diminish these functions leading to poor health habits and, subsequently, delay medication adherence.^7^, ^8^, ^9^ Well-being can have two aspects: objective and subjective well-being. Objective well-being mainly depends on the standard and level of living; these can be demonstrated through income, housing, occupation, education, food consumption, and nutrition.^10^ On the other hand, subjective well-being (SWB) or self-reported well-being refers to “people's cognitive and affective evaluations of their lives”.^11^ Cognitive evaluation is measured through the individual's overall life satisfaction, while affective evaluation is related to the individual's emotional experience.^12^

Given the rising costs of living, and increasing levels of stress in Lebanon, which have been exacerbated by the COVID-19 pandemic, and the current economic crisis,^13^, ^14^, ^15^ the relevance of positive psychology has become more pronounced. Additionally, more than half of the Lebanese population reported to have one or more chronic diseases.^16^ Managing multiple chronic diseases presents unique challenges, including greater health risks, increased healthcare utilization, and further financial burdens. In fact, in 2019, these chronic diseases accounted for the top causes of death, with ischemic heart disease being the leading cause, at 194 deaths per 100,000.^17^ The interplay of economic, humanistic, and clinical outcomes provides an opportunity to study how external stressors impact SWB and in return medication adherence. These factors make it essential to understand medication adherence behavior among this population.

Therefore, the objectives of this study were to explore the association between SWB and medication adherence among Lebanese individuals with multiple chronic diseases, and identify factors that influence medication adherence in this population. These investigations will serve as a preliminary step toward designing targeted interventions to enhance medication adherence behavior and overall health outcomes.

## 2 Methods

### 2.1 Study design

An exploratory, cross-sectional survey study was conducted to investigate the correlation between medication adherence and SWB.^18^ The study was carried out over three months, from December 2019 to February 2020. Data collection took place in six community pharmacies located in different areas of Beirut District in Lebanon. The selection of community pharmacies as primary sites over other healthcare outlets is based on their ability to offer a more demographic representative sample of the target population, their ease of access, and their cost-effectiveness. The questionnaire was distributed by an interviewer who administered it face-to-face to eligible participants.

### 2.2 Inclusion and exclusion criteria

Participants were included in the study, if they were Lebanese, above 18 years of age, self-reported to have at least one chronic disease, and taking at least one medication for their chronic disease for more than 12 months. The chronic diseases considered were hypertension, ischemic heart disease, heart failure, arrhythmia, diabetes mellitus, dyslipidemia, thyroid, stroke, epilepsy, osteoporosis, osteoarthritis/rheumatoid arthritis, asthma, chronic obstructive pulmonary disease, benign prostatic hyperplasia, and chronic kidney disease.^19^ Nonetheless, participants with previous mental/dementia disorders were excluded since the aim was to assess both the adherence and SWB of the participants under their volitional control.

### 2.3 Sample size calculation

Since there were no published global chronic disease prevalence studies specific to Beirut City, the sample size calculation was based on the highest estimated prevalence among selected chronic diseases, which was 38 % for cardiovascular diseases.^20^ Using the “Raosoft®” online sample size calculator and considering the Beirut adult population to account for 1,669,689, a total of 385 participants and above would provide a representative sample with a 5 % margin of error and a 95 % confidence level.

### 2.4 Questionnaire design

The questionnaire was divided into three main sections: (1) demographic socio-economic characteristics, (2) medication adherence scale, and (3) SWB scales. The questionnaire included a mix of self-developed questions for the first section, and standardized, reliable, and valid scales for the second and third sections.

#### 2.4.1 Demographic and socio-economic characteristics

This section was designed to collect information to form a brief background on the involved participants. It was developed after reviewing multiple pieces of literature to ensure the inclusion of culturally relevant characteristics.^19^^,^^21^, 22., 23 Henceforth, the first section included variables such as gender, age, marital status, education level, occupation, monthly personal income, health insurance, lifestyle, chronic diseases, medications taken, and the cost of chronic medications per month.

#### 2.4.2 Medication adherence scale

The second section assessed participants' medication adherence by using Adherence to Refills and Medication Scale Arabic Lebanese Version (ARMS-A).^24^^,^^25^ ARMS-A is the valid and reliable Arabic Version of the original ARMS.^24^^,^^25^ ARMS-A includes 12 items, each rated on a 4-point scale ranging from “none of the times”, computed as “1”, to “all of the times”, computed as “4”. The total sum score ranges from 12 to 48. To avoid acquisition bias, the 12th item was reverse-coded.^24^^,^^25^ ARMS-A is divided into two factors. Factor 1, is composed of six items (items 2, 6, 7, 8, 9, and 11), and is named “intentional non-adherence to taking medications”. Factor 2, is also composed of six items (1, 3, 4, 5, 10, and 12), and is labeled “unintentional non-adherence to taking medications and refilling prescriptions”.^25^ ARMS-A utilized a score of 12 as a cut-point value, with scores of 12 indicating high adherence and scores above 12 indicating low adherence. Thus, a lower score signifies better adherence.^24^^,^^25^

#### 2.4.3 Subjective well-being scales

SWB can be measured using numerous concepts. This study chose concepts including happiness, love of life, hope, and satisfaction with life because they represent distinct but complementary aspects of SWB, and they cover a broad range of emotional and cognitive evaluations of well-being.^18^^,^^26^, ^27^, ^28^, ^29^ Henceforth, the third section was comprised of four Arabic valid and reliable scales that were used to assess the SWB of participants,^18^ including The Arab Scale of Happiness 15-items (ASH),^26^ The Love of Life Scale 16-items (LLS),^27^ The Arab Hope Scale 8-items (AHS),^28^ and The Satisfaction with Life Scale 5-items (SWLS).^29^

ASH scale measures happiness in the Arabic language.^26^ ASH scale includes 15 brief statements, rated on a 5-point Likert scale from “Not at All”, rated as “1”, to “Very High”, rated as “5”. The total sum scores vary between 15 and 75. Higher scores indicate greater happiness.^26^ LLS scale measures the “positive attitude towards one's own life, a liking for it, and pleasurable attachment to it”. ^[^^27^^]^ LLS scale consists of 16 items, rated on a 5-point Likert scale from “No”, rated as “1”, to “Very Much”, rated as “5”. The total sum scores range from 16 to 80. Higher scores show stronger evidence of love for life.^27^ AHS scale measures hope, defined as goal-directed thinking where individuals perceive the capacity to find pathways to goals (i.e., pathways thinking) and the motivation to use these pathways (i.e., agency thinking).^28^ AHS scale consists of eight items, each rated on a 4-point scale from “Strongly disagree” valued as “1”, to “Strongly Agree” valued as “4”. The total sum scores range between 8 and 32. Higher scores reflect higher hope.^28^ SWLS scale evaluates overall life satisfaction.^29^ SWLS scale consists of five items, each rated on a 7-point scale with response choices ranging from “Strongly Disagree” valued as “1” to “Strongly Agree” valued as “7”. The total sum scores of the scale range from 7 to 35. Higher scores suggest greater satisfaction with life.^29^

### 2.5 Pre-testing process

The survey went through the pre-testing process before starting the data collection procedure. The process was divided into two stages: cognitive interviewing and pilot testing, both conducted by an individual who has experience working in both as a researcher and as a community pharmacist in Lebanon.

#### 2.5.1 Cognitive interviewing

This stage involved five participants from the target audience to ensure clarity and comprehensiveness of the survey questions. Participants were encouraged to think aloud while answering the questions to provide insights into their interpretation of the survey items. Based on their feedback, some rewording was done in the first section of the demographic and socioeconomic status questions to make them easier to understand. Also, the income variable was changed from continuous to categorical to maintain the privacy of the participants.

#### 2.5.2 Pilot testing

The next stage was pilot testing. It was conducted on thirty participants who represent the target population by using convenience sampling to check if the questions were properly interpreted. Following this stage, no modifications were made, and the survey was ready to be used for data collection. It is worth noting that individuals who participated in the pre-testing of the survey instrument were excluded from the final analysis.

### 2.6 Data collection procedure

#### 2.6.1 Sampling and pharmacy selection

Convenience sampling was carried out during the data collection process. A total of ten community pharmacies were initially approached to participate in this study. Out of these, six pharmacies agreed to participate, while the remaining four declined. The main reason for refusal was the concern of the owners toward potential daily operations disruption. The included community pharmacies were strategically selected to represent various areas within Beirut District, the Capital of Lebanon, based on the size and specific features of each pharmacy. This ensured the inclusion of a diverse range of participants' characteristics and socio-economic backgrounds. For example, three large retail pharmacies were included to theoretically represent high-class and wealthy individuals, whereas the other three reflected the residents from low-to-medium socio-economic backgrounds.

#### 2.6.2 Participant recruitment

On average, sixty-five participants were recruited from each community pharmacy (with a range of sixty to seventy participants per pharmacy). The data collection process occurred from December 2019 to the end of February 2020 during the first half of the weekdays (8 AM to 4 PM), which is when most individuals with chronic diseases pick up their chronic medications from the pharmacy. After receiving their medications and while leaving the pharmacy, participants were asked if they were taking any chronic medication and were briefly informed about the study and its purpose. To screen for eligibility, participants were informed about the exclusion and inclusion criteria and asked if they were willing to participate in the study. If the participant did not agree to be interviewed, they were allowed to decline without any ramifications to ensure that participation was entirely voluntary.

#### 2.6.3 Data collection process

The survey was administered through the interviewer-administration method by a researcher, who has experience working as a community pharmacist in Lebanon. The interviews were conducted in-person at the pharmacy. To maintain privacy and minimize any potential interruptions to the normal workflow of the pharmacy, a designated area within the pharmacy was used to introduce the study to participants and conduct the interviews. In this approach, questions were read out to the participants in the Arabic language to accommodate illiterate and/or low-literate individuals. Participants were responsible for interpreting and providing their own responses independently, without any aid from the interviewer. The average duration of the survey was around fifteen-to-twenty minutes. It is worth mentioning that participation in this study was voluntary with no distribution of incentives.

### 2.7 Data analysis

Collected data were entered, coded, and analyzed using Statistical Package for the Social Science (SPSS), version 23.

Descriptive statistics were employed to analyze all variables involved in the study. Continuous data were represented by median followed by Inter-Quartile Range (IQR), while categorical data were presented as counts (n) and percentage (%) occurrence of variables.

The overall standard Cronbach's alpha was measured on ARMS-A and SWB scales (ASH, LLF, AHS, and SWLS). Values between 0.7 and 0.9 were indicative of high internal consistency reliability.^30^^,^^31^

Spearman's Rho correlation coefficient statistic test was used to explore the association between the ARMS-A and SWB constructs (ASH, LLS, AHS, and SWLS). It was hypothesized that there would be no significant association between SWB and medication adherence, whereas an alternative hypothesis suggested a significant association between these variables. Further, a Chi-square statistical test (*χ*^2^) was employed to examine the correlation between ARMS-A score and all participants' characteristics. Variables that showed a *p* < 0.2 in the bivariate analyses were included in the logistic regression analysis.^32^^,^^33^

A forward LR binary logistic regression was conducted to identify factors that predict medication adherence among individuals with chronic diseases and on multiple chronic medications. The dependent variable, ARMS-A rating score, was regressed against all participants' characteristics identified as significant in earlier bivariate analyses.^32^^,^^33^ All categorical variables were recoded into dummy variables.

Findings were considered to be statistically significant when the *p* ≤ 0.05, with a confidence interval (CI) of 95 % (two-sided).

### 2.8 Ethical consideration

This research took approval from the Institutional Review Board of Beirut Arab University (2020-H-056-P-M-0380). Informed consent was signed only after thoroughly explaining the objectives and benefits of the current study to each participant. To ensure participant confidentiality, no personally identifiable information such as name, phone number, and address were collected. Code numbers were used instead of names and the collected data was securely stored in a closed locker.

## 3 Results

### 3.1 Participants enrollment

Out of 434 individuals initially screened, 425 of them were found eligible for this study. Moreover, only 400 of the eligible participants were included in the analysis because 21 of them refused to participate while the rest did not complete the instrument requested.

### 3.2 Participants characteristics

Participants were predominantly female (56.5 %), elderly (30.5 %), married (63.5 %), educated with a university degree (38.8 %), employed (48.8 %), and with a low monthly personal income ranging from $0 to $330 (35.8 %). Concerning lifestyle characteristics, participants had equal distribution between smokers and non-smokers with 44 % each, 85 % did not consume alcohol, and 60 % did not engage in regular physical activity. Further, over half of the participants did not follow any specific diet (60 %), and typically slept less than 7 h per night (53 %) (Table 1).

**Table 1:** Participants' Characteristics of the Studied Sample (*n* = 400).

Characteristics	n (%)
*Demographic and Socioeconomic Characteristics*	
Gender	
Male	174 (43.5)
Female	226 (56.5)
Age (years)	
18–24	13 (3.3)
25–34	45 (11.3)
35–44	45 (11.3)
45–54	64 (16.0)
55–64	111 (27.8)
≥ 65	122 (30.5)
Marital Status	
Single	76 (19.0)
Married	254 (63.5)
Divorced	14 (3.5)
Widowed	56 (14.0)
Educational Status	
University	155 (38.8)
High School	97 (24.3)
Middle School	52 (13.0)
Elementary School	61 (15.3)
Illiterate	35 (8.8)
Employment Status	
Employed	195 (48.8)
Unemployed	153 (38.3)
Retired	52 (13.0)
Occupation Status	
Health-Care Provider	76 (19.0)
Non Health-Care Provider	324 (81.0)

Monthly Personal Income (United States Dollars; conversion rate $1 = 1515 LBP)	
0–330	143 (35.8)
331–495	46 (11.5)
496–825	76 (19.0)
826–1650	91 (22.8)
1651 - 2770	37 (9.3)
> 2770	7 (1.8)
Medication Insurance	
Yes	265 (66.3)
No	135 (33.8)
Type of Insurance	
Insurance Company	31 (7.8)
State Employees	62 (15.5)
National Social Security Fund	145 (36.3)
Charity Centers	27 (6.8)
No Insurance	135 (33.8)
*Lifestyle Characteristics*	
Smoking Status	
Smoker	176 (44.0)
Non-Smoker	176 (44.0)
Ex-Smoker	48 (12.0)
Alcohol Status	
Alcohol Consumer	38 (9.5)
Non-Alcohol Consumer	337 (84.3)
Ex-Alcohol Consumer	25 (6.3)
Physical Activity Status (≥ 150 min/week)	
Yes	160 (40.0)
No	240 (60.0)
Diet Status (on any type of diet, e.g. low salt/fat, etc.)	
Yes	76 (19.0)
No	212 (53.0)
Sometimes	122 (28.0)
Sleeping Hours (per day)	
< 7 h	212 (53.0)
7–9 h	164 (41.0)
> 9 h	24 (6.0)
*Chronic Diseases Distribution*	
*Number of Chronic Diseases*	
1	119 (29.8)
2	111 (27.8)
≥ 3	170 (42.5)
Chronic Diseases	
Hypertension	232 (58.0)
Dyslipidemia	145 (36.3)
Coronary Artery Disease	34 (8.5)
Arrhythmia	111 (27.8)
Heart Failure	29 (7.3)
Diabetes Mellitus	155 (38.8)
Thyroid Disorders	50 (12.5)
Stroke	6 (1.5)
Epilepsy	20 (5.0)
Osteoporosis	52 (13.0)
Osteoarthritis	52 (13.0)
Asthma	29 (7.3)
Chronic Obstructive Pulmonary Disease	41 (10.3)
Benign Prostatic Hyperplasia	30 (7.5)
Chronic Kidney Disease	21 (5.3)
*Health System - Related Characteristics*	
Physicians Visits	
1	180 (45.0)
≥2	220 (55.0)
Hospitalization and/or Emergency Visits	
Zero	187 (46.7)
2	108 (27.0)
≥3	105 (26.3)
*Medication-Related Characteristics*	
Number of Chronic Medications	
1	66 (16.5)
2	65 (16.3)
3	66 (16.5)
4	55 (13.7)
≥ 5	148 (37.0)
Administration of Chronic Medications	
Self-Medications	360 (90.0)
Assisted by a family member or domestic helper	40 (10.0)
Route of Administration of Chronic Medications	
Oral	304 (76.0)
Injections	6 (1.5)
Inhalers	6 (1.5)
More than one route	84 (21.0)

Almost half of the participants (42.5 %) were managing three or more chronic diseases. Hypertension (58 %), diabetes mellitus (38.8 %), and dyslipidemia (36.3 %) were the most frequently observed chronic diseases, whereas stroke, epilepsy, and chronic kidney disease were the least with 1.5 %, 5 %, and 5.3 %, respectively. A notable fraction (37 %) of the participants were prescribed five or more chronic medications. The median monthly cost of these medications was $66. Regarding healthcare utilization, most of the participants (55 %) had two or more visits to any physician, whereas 46.7 % of the participants had zero hospitalization and/or emergency visit within the last year (Table 1).

### 3.3 ARMS-A 12-items distribution among the participants

ARMS-A scores were high with a median (IQR) = 16.00 (9.0) (Table S1, Supplementary Materials). This indicates low adherence among Lebanese individuals with chronic diseases. More importantly, only 26.5 % of the participants, 95 % CI, 0.222–0.311, were adherent to their chronic medications. The reliability analysis of the ARMS-A instrument showed that Cronbach's alpha was 0.859.

### 3.4 Subjective well-being constructs distribution among the participants

Participants' SWB was assessed using various scales (Table 2). The ASH had a median (IQR) of 46.00 (17.0) (Table S2, Supplementary Materials), the LLS total score had a median (IQR) of 54.00 (20.0) (Table S3, Supplementary Materials), the AHS total score had a median (IQR) of 24.00 (6.0) (Table S4, Supplementary Materials), and SWLS recorded a median (IQR) score of 24.00 (9.0) (Table S5, Supplementary Materials). After rating the SWLS scores, “slightly satisfied” and “satisfied” held the highest percentage, with 27.8 % and 32 %, respectively (Fig. S1, Supplementary Materials). Further, all four SWB scales demonstrated high internal consistency reliability with Cronbach's alpha of 0.925, 0.937, 0.923, and 0.866 for ASH, LLS, AHS, and SWLS, respectively (Table 2).

**Table 2:** Summary data of subjective well-being scales used in the studied sample (n = 400).

Subjective Well-Being Scales	Number of Items	Median (IQR)	Possible Range	Reliability (Cronbach's Alpha)
Arabic Scale of Happiness (ASH)				
ASH Total Score	15	46.00 (17.0)	15–75	0.925
Love of Life Scale (LLS)				
LLS Factor 1 (Positive Attitude Toward Life)	8	26.00 (10.0)	8–40	
LLS Factor 2 (Happy Consequences of Love of Life)	4	13.00 (6.0)	4–20	
LLS Factor 3 (Meaningful of Life)	4	14.00 (4.0)	4–20	
LLS Total Score	16	54.00 (20.0)	16–80	0.937
Arabic Hope Scale (AHS)				
AHS Factor 1 (Pathways)	4	12.00 (3.0)	4–16	
AHS Factor 2 (Agency)	4	12.00 (4.0)	4–16	
AHS Total Score	8	24.00 (6.0)	8–32	0.923
Satisfaction with Life Scale (SWLS)				
SWLS Total Score	5	24.00 (9.0)	5–35	0.866

### 3.5 Correlation of ARMS-A Score WITH SWB constructs' scores

Statistically significant negative correlations were observed between the ARMS-A score and the four SWB scores that range from r_s_ (400) = −0.160 to −0.272, *p* = 0.01 (Table 3). This shows that lower medication adherence (reflected in higher ARMS-A scores) is associated with lower SWB. In other words, as self-reported happiness, love of life, hope, and satisfaction with life among Lebanese individuals with chronic diseases decreases, their medication adherence tends to decrease as well.

**Table 3:** Correlation of ARMS-A score with swb constructs scores (n = 400).

	Subjective Well-Being Constructs			
	*ASH Score*	*LLS Score*	*AHS Score*	*SWLS Score*
ARMS-A Score	- 0.272 ⁎	- 0.160 ⁎	- 0.175 ⁎	- 0.211 ⁎

⁎ Correlation is statistically significant with a *p* = 0.01.

### 3.6 Regression of ARMS-A score with participants' characteristics

Several key variables significantly influenced medication non-adherence (Table 4). Within the demographic and socioeconomic status, only education, occupation, and income status were able to predict medication non-adherence. For example, the regression model showed that individuals with elementary/low education levels were found to be two times more likely to be non-adherent to their chronic medications, 95 % CI, 1.01–4.81, *p* = 0.045, when compared to those who were educated with a university degree. In contrast, participants earning over $2770 monthly had a significantly lower probability of non-adherence with an odds ratio (OR) = 0.062, 95 % CI, 0.01–0.38, *p* = 0.003 compared to those with earnings ranging from $0 to $330 per month. Lastly, individuals who self-identified themselves as healthcare providers were less likely to be non-adherent, OR = 0.422, 95 % CI, 0.21–0.84, *p* = 0.014, compared to those who did not (Table 4).

**Table 4:** Forward LR binary logistic regression predicting medication non-adherence behavior.

*Variables*	*B*	*SE*	*Wald*	*p-value*	*Exp (B)*	*95 % CI*
Educational Status (Elementary School) ^a^	+0.794	0.397	4.005	0.045	2.212	1.016–4.814
Monthly Personal Income (> $2770) ^b^	−2.774	0.929	8.911	0.003	0.062	0.010–0.386
Occupation Status (Health-care Provider) ^c^	−0.862	0.351	6.042	0.014	0.422	0.212–0.840
Chronic Disease (Diabetes Mellitus) ^d^	−0.525	0.251	4.360	0.037	0.592	0.362–0.968
Diet Status (No) ^e^	+0.500	0.251	3.969	0.046	1.649	1.008–2.696
Number of Hospitalization/ Emergency Visit (Two) ^f^	+1.193	0.321	13.84	0.001	3.296	1.759–6.178
Number of Hospitalization/ Emergency Visit(≥Three) ^g^	+0.998	0.326	9.370	0.002	2.713	1.432–5.140

Abbreviations: B: Coefficient for the constant (Intercept); S.E: Standard Error around the coefficient for the constant; Wald: Wald Chi-Square Statistical Test; Exp (B): Odds Ratio for the predictors; CI: Confidence Interval.

Notes

a. Reference category: Educated with a university degree.

b. Reference category: $0–330 monthly personal income.

c. Reference category: Non-health-care provider.

d. Reference category: Not diagnosed diabetes mellitus.

e. Reference category: On any type of diet (Yes).

f. Reference category: Zero hospital and/or emergency visit.

g. Reference category: Zero hospital and/or emergency visit.

Among all the lifestyle characteristics that were included in this study, diet status was the only variable that was able to predict medication non-adherence. The regression model showed that individuals who did not follow any healthy diet lifestyle had a higher probability of being non-adherent, OR = 1.5, 95 % CI, 1.01–2.69, *p* = 0.046 compared to those who stick to a healthy diet lifestyle. Further, the model predicted that individuals with diabetes mellitus were less likely to be non-adherent to their chronic medications, OR = 0.592, 95 % CI, 0.36–0.96, *p* = 0.037 when set against individuals without diabetes mellitus (Table 4).

Out of the health system-related characteristics, the number of hospitalizations and/or emergency visits seems to act as a predictor of medication non-adherence. The model showed that individuals who had 2 and ≥ 3 hospital admissions and/or emergency visits in the last 12 months were around 3 times more probably to be non-adherent to their chronic medications when differentiated from individuals who had zero hospital admission and/or emergency visit, 95 % CI, 1.75–6.17, and 1.43–5.14, *p* = 0.001, and *p* = 0.002, respectively (Table 4).

## 4 Discussion

The study results shed light on how SWB impacted medication adherence among Lebanese individuals with multiple chronic diseases. Given the ongoing economic crisis in Lebanon, this research explored the complex relationship between economic stressors, psychological well-being, and health-related outcomes. To our knowledge, no prior studies have specifically examined this interplay within the socio-economic conditions of Lebanon. Additionally, the study identified key predictors of medication adherence which offered a deeper understanding of the barriers and challenges to adherence in this population.

### 4.1 Distribution of chronic diseases among participants

Nearly half of the participants (42.5 %) reported managing three or more chronic diseases concurrently, while the rest had one to two chronic diseases, with 29.8 % and 27.8 %, respectively. Such distribution mirrors the findings of Ramia and colleagues.^34^ They reported that a large portion of Lebanese individuals (47.6 %) were diagnosed with three or more chronic diseases simultaneously.^34^ Previous research indicated that individuals with multiple chronic diseases face significantly higher health risks, including premature death, increased hospital admissions, prolonged hospital stays, poorer clinical outcomes, and diminished quality of life, compared to those who manage a single chronic disease.^35^ Further, these individuals are more prone to experience mental health challenges, such as anxiety and depression.^36^ Collectively, these factors contribute to the complexities Lebanese individuals encounter in maintaining medication-taking behavior.^35^^,^^37^ Moreover, this study's findings pinpointed hypertension, diabetes mellitus, and dyslipidemia as the predominant chronic diseases. These results align with those reported by Sibai and colleagues.^38^ This suggests that cardiovascular and endocrine system-related diseases are prevalent among the Lebanese population, reflecting the impact of poor and unhealthy lifestyle behaviors.

### 4.2 Chronic medication-related characteristics among participants

One hundred forty-eight participants (37 %) were prescribed five or more chronic medications to manage their chronic diseases. Ramia and colleagues reported around 16 % of their study cohort was also taking five or more chronic medications daily.^34^ Such pattern indicates a prevalence of polypharmacy among the Lebanese population. Unfortunately, polypharmacy can be troublesome and introduces numerous challenges, including elevated risks of drug duplication, drug-drug interactions, adverse drug reactions, and higher healthcare costs.^39^^,^^40^ Consequently, polypharmacy can notably complicate medication management for individuals with chronic diseases, which may potentially lead to medication non-adherence.^39^ This suggests the need for a comprehensive medication review, patient education, and other medication management strategies to reduce adverse events and boost medication adherence.^41^^,^^42^

In addition, the cost of chronic medications per month has a median (IQR) = $66 (132). Such a cost reflects a significant financial burden, especially considering that a large portion of the participants (36 %) reported a low monthly personal income that ranges from $0 to $330. Given the nature of chronic diseases which require continuous management and generally can not be cured, this financial burden can substantially strain individuals' ability to afford their chronic medications and sustain long-term medication adherence.^43^

### 4.3 Distribution of ARMS-A rating score among participants

The ARMS-A had a median (IQR) of 16.00 (9.0) which indicates that there is a trend toward low medication adherence among the Lebanese population. In fact, three-quarters of the study's participants were non-adherent to the chronic medications needed to manage their chronic diseases, leaving only 106 participants (26.5 %) to be classified as adherent. According to Al-Hajj and colleagues, only 42.6 % out of the 148 participants were adherent.^33^ Similarly, Mroueh and colleagues reported that out of 245 Lebanese individuals with diabetes mellitus, 78 (31.8 %) and 167 (68.2 %) participants were classified to be adherent and non-adherent to their chronic medications, respectively.^44^ Consequently, poor medication adherence is perceived as a significant challenge in managing non-communicable chronic diseases.

### 4.4 Subjective well-being and medication adherence

The present study showed that reduced medication adherence (reflected by elevated ARMS-A scores) correlates with a decrease in SWB. Such results match earlier research findings.^45^, ^46^, ^47^ For example, Cuffee and colleagues assessed the correlation between happiness and medication adherence scores using Lyubomrisky & Lepper and Morisky scales, respectively in a cohort of 573 African American hypertensive women.^45^ The results revealed that women with higher happiness scores exhibited better medication adherence attitudes compared with lower counterparts after controlling other co-variants.^45^ Similarly, a strong positive correlation was observed between medication adherence and hope with *r* = 0.893, *p* < 0.05.^46^ In the context of Lebanon, a study revealed that global satisfaction with treatment, convenience, and effectiveness are predominant factors in boosting medication adherence.^47^ In return, individuals who adhere to their chronic medications reported an improved quality of life compared to those who do not.^47^ To address the low SWB observed, accessible and affordable mental health screening, counseling services, and stress management programs are highly warranted.^48^, ^49^, ^50^ Moreover, community-based initiatives, including support groups and social engagement activities can significantly boost SWB and medication adherence of individuals with chronic diseases.^48^, ^49^, ^50^

### 4.5 Predictors of medication adherence

The current study findings reflect that most demographic and socioeconomic factors including gender, age, marital status, employment status, and health insurance status, did not predict the medication adherence behavior among participants. Likewise, Al-Hajje and colleagues, stated that socio-demographic variables did not significantly influence adherence among Lebanese individuals with chronic diseases.^33^ Yet, in this study, the regression analysis showed that education plays a key role in medication adherence. Specifically, individuals with only elemental education were two times more likely to be non-adherent compared to those with university degrees. Pandey and colleagues mirror this analysis by highlighting that educational status seems to be an essential determinant of medication adherence.^51^ This necessitates the need for tailored and targeted counseling and educational sessions that match the individual's level of understanding.^52^^,^^53^

Further, financial status revealed as a significant predictor of medication adherence. In particular, wealthy individuals, with a monthly personal income ≥ $2700, showed higher adherence levels compared to those with low monthly personal income ranging from $0–330. A systemic review confirms that individuals with “lower self-paying constraints” had greater adherence compared to those who were financially constrained.^54^ In parallel, another study showed that financial strain is correlated with poor self-rated health and low adherence.^55^ Such findings pinpoint the influence of economic constraints, that the Lebanese population currently experiencing, on both SWB and medication adherence. This emphasizes the critical need for healthcare policymakers, including the Ministry of Public Health in Lebanon, to develop and implement a map with better health policies and regulations that address the financial burden.

Lastly, the regression model showed that healthcare providers have higher adherence rates, mainly because of their high health literacy levels. Providers can assess, understand, and use the acquired knowledge to make proper health decisions; hence, they persist in taking their chronic medications.^56^

Within the regression model, diet emerged as the sole lifestyle factor that predicted medication non-adherence. Individuals not following a healthy diet were found to be 1.5 times more likely to be non-adherent compared to those maintaining a nutritious diet. Such a correlation conflicts with some previous research^57^ and aligns with others.^58^ For instance, a study involving 417 Korean individuals demonstrated that medication adherence was significantly associated with numerous lifestyle modifications including low-salt intake, diet, exercise, and smoking cessation, OR = 11.7, 95 % CI, 1.5–91.3.^57^ However, another study revealed that 68.2 % of individuals with hypertension were not adherent to their chronic medications, and 45.1 % had a high salt intake.^58^ Notably, almost half of the participants experienced elevated blood pressure levels because of excessive salt consumption.^58^ This could imply that dietary habits have a more immediate impact on medication adherence and/or that other lifestyle factors were not as strongly practiced among the participants. Therefore, early identification of individuals at risk of non-adherence to both chronic medications and lifestyle modifications is vital to speed up the implementation of motivational and educational programs that are designed to enhance overall adherence.

Among chronic disease management, diabetes mellitus demonstrated a significant indicator of medication adherence. Individuals with diabetes were more likely to follow their medication regimen compared to their counterparts. This finding diverges from other international studies where non-adherence among individuals with diabetes remains a challenge.^59^^,^^60^ For example, Wabe and colleagues showed that 58.2 % of Ethiopian individuals with diabetes failed to achieve proper glycemia control because of non-adherence behavior, primarily due to financial constraints and fear of adverse drug reactions.^59^ Similarly, another study found that more than half (54.4 %) of individuals with diabetes were non-adherent, which is attributed to forgetfulness, financial issues, and symptom disappearance.^60^

For health system-related factors, the number of hospitalizations and/or emergency visits significantly predicts non-medication adherence. Individuals who experienced two or more visits within the last year were found to be three times more likely to be non-adherent to their chronic medication than those with lower or no healthcare utilization. This pattern aligns with a meta-analysis by Mongkhon and colleagues, which highlighted that 1–10 % of outpatient hospital admissions were due to medication non-adherence.^61^ These insights emphasize the critical impact of non-adherence on healthcare utilization and pinpoint the need for targeted interventions to support individuals with frequent healthcare engagements.

The findings of this study have critical implications. Clinically, recognizing SWB as a crucial factor in medication adherence highlights the necessity for tailored interventions that address both the psychological and medical needs of individuals with chronic diseases. Healthcare providers, particularly pharmacists, are advised to integrate mental health assessment and consider social determinants of health in their daily routine care to improve medication adherence. Further, policymakers are encouraged to develop strategies that alleviate the financial burden of chronic medications. This can be accomplished by improving healthcare insurance coverage, supporting the use of generic medications manufactured by Lebanese pharmaceutical companies, and collaborating with non-governmental and charity health organizations. Future studies should focus on developing and implementing culturally-tailored interventions, led by pharmacists, to address the specific needs of the Lebanese population, and consequently, enhance medication adherence and overall health outcomes. By addressing the unique challenges faced by this population, this research paves the way for more effective and contextually relevant healthcare strategies.

### 4.6 Strengths and limitations

It is worth mentioning that cross-sectional survey design does not permit causal inferences as both exposure and outcome were simultaneously assessed. However, this is one of the early studies that explored the intersection of SWB and medication adherence for Lebanese individuals during the economic crisis. Further, the study was carried out in Beirut through convenience sampling rather than across the entire country; hence, limiting the generalizability of findings to other settings. Yet, efforts to mitigate this limitation included collecting data from different areas in Beirut City, the Capital of Lebanon, with different participants' characteristics and backgrounds. Additionally, reliance on self-report measures while conducting interviewer-administered questionnaires to accommodate all participants might lead to an overestimation of medication adherence and SWB because both are considered socially desirable behaviors. However, in an attempt to reduce this bias, the survey was free from any identifying information so that the participants could express themselves freely and honestly without any concerns.

## 5 Conclusion

A low prevalence of medication adherence among Lebanese individuals with chronic diseases highlighted the complex interrelation between socioeconomic, psychological, and health system-related factors. Further, lower adherence levels were correlated with reduced subjective well-being, including happiness, love of life, hope, and satisfaction with life. This underscores the need for holistic chronic disease management strategies that integrate both clinical and psychosocial elements.

## Funding

The authors declare that no funds, grants, or other support were received during the preparation of this manuscript.

## Ethical approval

The study was performed in line with the principles of the Declaration of Helsinki. Ethical approval was obtained from the Institutional Review Board of Beirut Arab University (# 2020-H-056-P-M-0380).

## Consent to participate

Informed consent was attained from all the participants included in the study.

## Consent for publication

The undersigned qualify as authors of this manuscript and confirm that nobody who qualifies for authorship has been excluded. The authors agree to submit this manuscript to Exploratory Research in Clinical and Social Pharmacy, and if accepted, to its publication in this journal. The authors assure that this manuscript is original, and it is not under consideration by another journal, nor it has been previously published.

## CRediT authorship contribution statement

**Mohamad Ismail:** Writing – review & editing, Writing – original draft, Visualization, Formal analysis, Data curation, Conceptualization. **Mayssah El-Nayal:** Writing – review & editing, Conceptualization. **Souraya Domiati:** Writing – review & editing, Conceptualization.

## Declaration of competing interest

The authors declare no competing interests.

## Data Availability

Some data are added to the supplementary materials.

## Appendix A Supplementary data

Supplementary material Image 1
