# Supplementary material for: Exploring the impact of subjective well-being on medication adherence: A cross-sectional study among individuals with multiple chronic diseases
Source: Explor Res Clin Soc Pharm. 2024 Aug 25;15:100496. doi: 10.1016/j.rcsop.2024.100496 (PMC11403057; doi:10.1016/j.rcsop.2024.100496)
Supplement: Supplementary file 1 — Supplementary material [file mmc1.docx]

**Supplementary Materials:**

Table S1: ARMS-A 12-Items Distribution of the Studied Sample (n=400)

| **ARMS-A**  **Items** | **n (%)** | | | |
| --- | --- | --- | --- | --- |
|  | ***None*** | ***Some*** | ***Most*** | ***All*** |
| 1. How often do you forget to take your medicine? | 238 (59.5) | 138 (34.5) | 17 (4.3) | 7 (1.8) |
| 2. How often do you decide not to take your medicine? | 278 (69.5) | 94 (23.5) | 21 (5.3) | 7 (1.8) |
| 3. How often do you forget to get prescriptions filled? | 260 (65.0) | 104 (26.0) | 28 (7.0) | 8 (2.0) |
| 4. How often do you run out of medicine? | 240 (60.0) | 120 (30.0) | 27 (6.8) | 13 (3.3) |
| 5. How often do you skip a dose of your medicine before you go to the doctor? | 293 (73.3) | 80 (20.0) | 20 (5.0) | 7 (1.8) |
| 6. How often do you miss taking your medicine when you feel better? | 268 (67.0) | 70 (17.5) | 48 (12.0) | 14 (3.5) |
| 7. How often do you miss taking your medicine when you feel sick? | 305 (76.3) | 71 (17.8) | 17 (4.3) | 7 (1.8) |
| 8. How often do you miss taking your medicine when you are careless? | 273 (68.3) | 86 (21.5) | 30 (7.5) | 11 (2.8) |
| 9. How often do you change the dose of your medicines to suit your needs? | 259 (64.8) | 104 (26.0) | 28 (7.0) | 9 (2.3) |
| 10. How often do you forget to take your medicine when you are supposed to take it more than once a day? | 249 (62.3) | 192 (32.3) | 17 (4.3) | 5 (1.3) |
| 11. How often do you put off refilling your medicines because they cost too much money? | 271 (67.8) | 92 (23.0) | 25 (6.3) | 12 (3.0) |
| 12. How often do you plan ahead and refill your medicines before they run out? *** | 230 (57.5) | 113 (28.3) | 44 (11.0) | 13 (3.3) |

* ARMS-A 12 is a reverse-coded item

Table S2: ASH 15-Items Distribution of the Studied Sample (n=400)

| **ASH**  **Items** | **n (%)** | | | | |
| --- | --- | --- | --- | --- | --- |
|  | **Not at all** | **A Little** | **Moderate** | **High** | **Very High** |
| 1. I have an overall sense of wellbeing. | 26 (6.5) | 79 (19.8) | 172 (43.0) | 93 (23.3) | 30 (7.5) |
| 2. I am happy with my life style. | 50 (12.5) | 60 (15.0) | 159 (39.8) | 90 (22.5) | 41 (10.3) |
| 3. I love life. | 23 (5.8) | 44 (11.0) | 112 (28.0) | 139 (34.8) | 82 (20.5) |
| 4. My life has meaning. | 31 (7.8) | 35 (8.8) | 111 (27.8) | 150 (37.5) | 73 (18.3) |
| 5. I am as happy now as when I was younger. | 189 (47.3) | 75 (18.8) | 88 (22.0) | 33 (8.3) | 15 (3.8) |
| 6. I feel good about my personal life. | 53 (13.3) | 53 (13.3) | 152 (38.0) | 93 (23.3) | 49 (12.3) |
| 7. My daily life is full of pleasant experiences. | 69 (17.3) | 101 (25.3) | 144 (36.0) | 56 (14.0) | 30 (7.5) |
| 8. I feel relaxed and free from tension. | 101 (25.3) | 113 (28.3) | 119 (29.8) | 47 (11.8) | 20 (5.0) |
| 9. I enjoy what I do. | 51 (12.8) | 83 (20.8) | 113 (28.3) | 103 (25.8) | 50 (12.5) |
| 10. I feel optimistic about the future. | 66 (16.5) | 87 (21.8) | 131 (32.8) | 72 (18.0) | 44 (11.0) |
| 11. I feel full of vitality and energy. | 69 (17.3) | 86 (21.5) | 136 (34.0) | 76 (19.0) | 33 (8.3) |
| 12. I feel that I am successful. | 45 (11.3) | 67 (16.8) | 136 (34.0) | 98 (24.5) | 54 (13.5) |
| 13. I feel my mental state is excellent. | 67 (16.8) | 68 (17.0) | 153 (38.3) | 68 (17.0) | 44 (11.0) |
| 14. I am satisfied with my life. | 44 (11.0) | 47 (11.8) | 124 (31.0) | 116 (29.0) | 69 (17.3) |
| 15. I have friendly feelings towards others. | 11 (2.8) | 23 (5.8) | 41 (10.3) | 150 (37.5) | 175 (43.8) |

Table S3: LLS 16-Items Distribution of the Studied Sample (n=400)

| **LLS**  **Items** | **n (%)** | | | | | |  |
| --- | --- | --- | --- | --- | --- | --- | --- |
|  | **No** | **A Little** | **Moderate** | **Much** | **Very Much** | | |
| 1. Life is full of pleasures. | 34 (8.5) | 85 (21.3) | 139 (34.8) | 104 (26.0) | | 38 (9.5) |  |
| 2. There are many things that make me love life. | 29 (7.3) | 75 (18.8) | 138 (34.5) | 119 (29.8) | | 39 (9.8) |  |
| 3. Love of life adds to its beauty. | 30 (7.5) | 64 (16.0) | 113 (28.3) | 142 (35.5) | | 51 (12.8) |  |
| 4. Life deserves to be loved. | 31 (7.8) | 59 (14.8) | 123 (30.8) | 135 (33.8) | | 52 (13.0) |  |
| 5. Love of life makes me happy. | 28 (7.0) | 73 (18.3) | 132 (33.0) | 132 (33.0) | | 35 (8.8) |  |
| 6. Life seems beautiful & wonderful to me. | 45 (11.3) | 81 (20.3) | 136 (34.0) | 104 (26.0) | | 34 (8.5) |  |
| 7. I look at life from its beautiful side. | 26 (6.5) | 87 (21.8) | 109 (27.3) | 137 (34.3) | | 41 (10.3) |  |
| 8. Love of life gives me hope. | 29 (7.3) | 73 (18.3) | 121 (30.3) | 136 (34.0) | | 41 (10.3) |  |
| 9. I would like to have a long life to achieve what I hope for. | 45 (11.3) | 57 (14.3) | 112 (28.0) | 123 (30.8) | | 63 (15.8) |  |
| 10. Love of life brings me satisfaction. | 41 (10.3) | 73 (18.3) | 135 (33.8) | 121 (30.3) | | 30 (7.5) |  |
| 11. Life is beautifully meaningful. | 30 (7.5) | 60 (15.0) | 115 (28.8) | 141 (35.5) | | 54 (13.5) |  |
| 12. Life is a treasure we should guard. | 33 (8.3) | 65 (16.3) | 121 (30.3) | 129 (32.3) | | 52 (13.0) |  |
| 13. Life is a blessing whose value we should appreciate. | 15 (3.8) | 47 (11.8) | 82 (20.5) | 186 (46.5) | | 70 (17.5) |  |
| 14. I realize that my existence in this life had great meaning. | 17 (4.3) | 54 (13.5) | 99 (24.8) | 154 (38.5) | | 76 (19.0) |  |
| 15. I always have a wonderful feeling of loving life. | 38 (9.5) | 69 (17.3) | 122 (30.5) | 130 (32.5) | | 41 (10.3) |  |
| 16. I like to be optimistic about life. | 18 (4.5) | 64 (16.0) | 90 (22.5) | 150 (37.5) | | 78 (19.5) |  |

Table S4: AHS 8-Items Distribution of the Studied Sample (n=400)

| **AHS**  **Items** | **n (%)** | | | |
| --- | --- | --- | --- | --- |
|  | ***Definitely False*** | ***Mostly False*** | ***Mostly True*** | ***Definitely True*** |
| 1. I can think of many ways to get out of a jam. | 15 (3.8) | 34 (8.5) | 214 (53.5) | 137 (34.3) |
| 2. I energetically pursue my goals. | 13 (3.3) | 40 (10.0) | 195 (48.8) | 152 (38.0) |
| 3. There are lots of way around any problem. | 12 (3.0) | 31 (7.8) | 201 (50.3) | 156 (39.0) |
| 4. I can think of many ways to get the things in life that are important to me. | 14 (3.5) | 57 (14.3) | 226 (56.5) | 103 (25.8) |
| 5. Even when others get discouraged, I know I can find a way to solve the problem. | 12 (3.0) | 63 (15.8) | 219 (54.8) | 106 (26.5) |
| 6. My past experiences have prepared me well for my future. | 21 (5.3) | 74 (18.5) | 186 (46.5) | 119 (29.8) |
| 7. I have been pretty successful in life. | 20 (5.0) | 86 (21.5) | 215 (53.8) | 79 (19.8) |
| 8. I meet the goals that I set for myself. | 18 (4.5) | 96 (24.0) | 210 (52.5) | 76 (19.0) |

Table S5: SWLS 5-Items Distribution of the Studied Sample (n=400)

| **SWLS**  **Items** | **n (%)** | | | | | | |
| --- | --- | --- | --- | --- | --- | --- | --- |
|  | ***Strongly Disagree*** | ***Disagree*** | ***Slightly Disagree*** | ***Neutral*** | ***Slightly Agree*** | ***Agree*** | ***Strongly Agree*** |
| 1. In most ways my life is close to my ideal. | 10(2.5) | 36(9.0) | 31(7.8) | 51(12.8) | 107(26.8) | 131(32.8) | 34(8.5) |
| 2. The conditions of my life are excellent. | 23(5.8) | 52(13.0) | 37(9.3) | 53(13.3) | 125(31.3) | 88(22.0) | 22(5.5) |
| 3. I am satisfied with my life. | 11(2.8) | 35(8.8) | 22(5.5) | 38(9.5) | 97(24.3) | 163(40.8) | 34(8.5) |
| 4. So far I have gotten the important thing I want in life. | 14(3.5) | 46(11.5) | 39(9.8) | 47(11.8) | 105(26.3) | 120(30.0) | 29(7.3) |
| 5. If I could live my life over, I would change almost nothing. | 12(3.0) | 42(10.5) | 58(14.5) | 19 (4.8) | 111(27.8) | 128(32.0) | 30(7.5) |

Figure S1: SWLS 5-Items Rating Score in the Studied Sample (n=400)
